# Positive Lymph Node Ratio as a new prognostic score in Geriatric patients with operated gastric cancer

**DOI:** 10.1016/j.heliyon.2024.e38809

**Published:** 2024-10-02

**Authors:** Omer Acar, Onur Yazdan Balcik, Muslih Urun, Tugay Avci, Mustafa Sahbazlar, Atike Pinar Erdogan

**Affiliations:** a Manisa Celal Bayar University, Medical Oncology Department, Manisa, Turkey; b Mardin Training and Research Hospital Medical Oncology, Mardin, Turkey; c Van YuzuncuYil University, Medical Oncology Department, Van, Turkey

**Keywords:** PLNR score, Gastric cancer, Overall survival

## Abstract

**Objective:**

The pivotal prognostic determinant for recurrence and survival in surgically treated gastric cancer (GC) patients remains the lymph node status. Despite the adoption of D2 lymph node dissection as the standard approach in recent years, its association with increased morbidity in elderly patients raises concerns. This study aims to explore the prognostic significance of the Positive Lymph Node Ratio (PLNR) score in the context of recurrence and survival among elderly patients with surgically treated GC.

**Material and method:**

A retrospective review of files about surgically treated patients with GC was conducted. The prognostic impact of the PLNR score on overall survival (OS) was assessed through Receiver Operating Characteristic (ROC) analysis.

**Results:**

The cut-off value for the PLNR, determined through ROC analysis, was identified as 0.138. This value serves as a crucial threshold, as it distinguishes patients with a higher risk of poor outcomes. Patients with a PLNR score of 0.138 or below exhibited a median OS of 111 months, whereas those with a PLNR score above 0.138 had a significantly lower median OS of 22 months (p = 0.004).

**Conclusion:**

Our findings revealed that the PLNR emerged as an independent predictor of survival and recurrence in patients undergoing GC resection.However, it's important to note that while valuable, the PLNR system has limitations. It does not encompass the T stage, a key factor in cancer staging. Therefore, it cannot be a direct substitute for the comprehensive information TNM staging provides. It should be used as a supplementary tool in predicting prognosis, particularly in elderly patients unsuitable for standard lymph node dissection.

## 1 Introduction

Gastric cancer (GC) is a global health concern, ranking as the fifth most prevalent cancer worldwide and standing as the third leading cause of cancer-related mortality [1]. With the aging population on the rise, a significant 60 % of patients receive a diagnosis of GC over the age of 65 [2]. The incidence of GC is notably higher in far eastern countries than in the Western population. However, the success of screening programs in Far Eastern countries has played a crucial role in the early detection of GC, offering hope for effective treatment through endoscopic resection. In contrast, in Western countries, GC tends to be diagnosed at advanced stages [3,4]. While numerous staging systems exist, TNM and AJCC (American Joint Committee on Cancer) stand out as preeminent tools for prognostic assessment in gauging cancer progression [5]. The TNM staging system, being the most widely employed, indicates that in cases without distant metastasis, endoscopic resection offers a potential cure when the tumor is at the T1 stage and node-negative.

For resectableGC at the T2 stage and beyond, the standard of care involves surgical resection, preferably incorporating perioperative chemotherapy and D2 lymph node dissection [6]. The treatment principles for elderly patients are consistent with those for non-elderly patients. However, it's crucial to remember that the ECOG performance status, frailty, and comorbidities such as hypertension and diabetes exert a significant impact on both treatment strategies and prognosis [7]. Elderly patients are more prone to postoperative wound care complications [8]. The prognosis in GC is contingent upon the surgical procedure undertaken and the extent of lymph node dissection. According to AJCC guidelines, a minimum of 16 lymph nodes should be dissected for an accurate prognosis.In elderly patients, the curtailed lymph node dissection, prompted by concerns about postoperative morbidity, may not accurately reflect the actual nodal involvement and subsequent prognosis.For this reason, many authors have introduced the Positive Lymph Node Ratio (PLNR) in recent years as a supplementary measure to TNM staging. The PLNR, characterized by the ratio of positive lymph nodes to the total number of lymph nodes removed during curative resection, has been proposed as a novel prognostic score for individuals with GC [[9], [10], [11], [12]]. It does not aim to replace TNM staging but rather to enhance its predictive power, particularly in elderly patients unsuitable for standard lymph node dissection. In this study, our objective was to assess the prognostic relevance of PLNR in elderly GC patients undergoing surgery, particularly in the ongoing debate surrounding the adequacy of nodal classification in current staging systems.

## 2 Material and method

This study, conducted and designed in accordance with the Declaration of Helsinki and Good Clinical Practices, was approved by the ethics committee of ManisaCelal Bayar University (March 15, 2023 and No. 1745), underscoring our commitment to ethical research. As a retrospective study, no additional informed consent was obtained from the patients. The medical records of patients who underwent GC surgery at two medical centers, ManisaCelal Bayar University Hafsa Sultan Hospital and Van YuzuncuYil University Hospital, were reviewed between January 2002 and December 2021. The data collection process was thorough, ensuring the inclusion of patients who consistently attended follow-up visits. The inclusion criteria encompassed patients aged 65 and above who had undergone surgery following a diagnosis. Exclusion criteria comprised patients under 65 years of age and those who did not undergo surgery due to either metastatic gastric cancer or concurrent comorbidities at the time of diagnosis. Various clinical and pathological characteristics, including age, gender, performance status, smoking history, BMI (body mass index), comorbidity, tumor marker levels at the time of diagnosis, type of operation, surgical margin status, site of tumor involvement, histological subtype, HER2 status, tumor grade, neural invasion, vascular invasion, treatment received (neoadjuvant/adjuvant chemotherapy, adjuvant radiotherapy), TNM stage, metastatic lymph nodes involved, and total lymph nodes removed were recorded.

Our study primarily focused on two critical endpoints: overall survival (OS) and recurrence-free survival, which are crucial in GC surgery. We also evaluated the PLNR, the ratio of metastatic lymph nodes retained to the total number of lymph nodes removed, and its impact on overall survival (OS) through ROC analysis based on the patients' PLNR scores.

## 3 Statistical analysis

Statistical analysis was conducted using SPSS 15.0 for Windows. Categorical variables were presented as numbers and percentages, while numerical variables were described using mean, standard deviation, minimum, maximum, and median values. Survival rates were calculated through Kaplan-Meier Analysis. Cox regression analysis was utilized to analyze risk factors, and the statistical significance level was set at p < 0.05.

## 4 Results

The study encompassed 184 patients from two centers, with 66 % male. The median age at diagnosis was 71 years (range: 65–86). The median body mass index was 24.3 (range: 17–39), and eighty-two percent of the participants exhibited an Eastern Cooperative Oncology Group (ECOG) performance score of 0–1. Twenty-five percent of patients underwent D2 lymph node dissection. The histologic subtype characterized by signet-ring cells was observed in 21 % of cases. Thirty-nine percent of cases exhibited a poorly differentiated carcinoma. Half of the patients were classified as TNM stage 3. The positivity rate for HER2 receptors stood at 18.5 %. The mean number of lymph nodes removed was 26.4 (range: 3–65), with a mean of 6.5 (range: 0–42) metastatic lymph nodes retained, resulting in a median PLNR of 0.24 (range: 0–1) (Table 1).

**Table 1:** Demographic and disease characteristics of the patient group.

N = 184		**Total**	
		N	%
**Gender**	Female	61	33.2
	Male	123	66.8
**Age mean**.±SD (Min-Max)		70.8 ± 5.4 (64–86)	
**Comorbidity**	No	86	52.8
	Yes	77	47.2
**Smoking**	Never	99	58.9
	Smoker	69	41.1
**Surgical margin**	Negative	166	91.7
	Positive	15	8.3
**Surgical type**	Subtotal gastrectomy + d2	10	5.5
	Subtotal gastrectomy + d1	73	40.3
	Total gastrectomy + d1	63	34.8
	Total gastrectomy + d2	35	19.3
**Grade**	Well differentiated	5	3.6
	Moderately differentiated	80	57.6
	Poorly differentiated	54	38.8
**Diagnosis CEA** Median (Min-Max)		2.5 (0–110)	
**Diagnosis Ca199** Median (Min-Max)		12.85 (0.30–1710)	
**Ca125 median** (Min-Max)		11 (1.6–72.5)	
**Neural invasion**	No	57	34.5
	Yes	108	65.5
**Vascular invasion**	No	60	35.9
	Yes	107	64.1
**Removed LN** Mean ± SD (Min-Max)		26.4 ± 13.4 (3–65)	
**Metastatic LN** Mean ± SD (Min-Max)		6.5 ± 9.3 (0–42)	
**PLNR** Mean ± SD (Min-Max)		0.24 ± 0.29 (0–1)	
**TNM**	Stage 0	3	1.7
	Stage 1	15	8.3
	Stage 2	63	35.0
	Stage 3	91	50.6
	Stage 4	8	4.4
**HER-2 receptor**	Negative	88	81.5
	Positive	20	18.5
**BMI** Mean.±SD (Min-Max)		24.3 ± 4.2 (16.7–39.6)	
**ECOG**	Ecog 0	92	50.3
	Ecog 1	58	31.7
	Ecog 2	28	15.3
	Ecog 3-4	5	2.7
**NeoadjuvantCT**	No	137	74.5
	Yes	47	25.5
**AdjuvantCRT**	No	129	70.1
	Yes	55	29.9
**Adjuvantchemotherapy**	No	43	23.4
	Yes	141	76.6

**XELOX**: Capecitabine plus oxaliplatin**FLOT**: Fluorouracil plus oxaliplatin plus docetaxel **DCF:** Docetaxel plus cisplatin plus fluorouracil.

**CT**: Chemotherapy **CRT**: Chemoradiotherapy**ECOG**: Eastern cooperative oncology group **BMI**: Body max index.

Surgical margin positivity, neural invasion, vascular invasion rates, the number of metastatic lymph nodes, and mean PLNR were significantly higher in patients with recurrence compared to those without recurrence (p = 0.011, p = 0.008, p = 0.008, p < 0.001, p = 0.002, p = 0.007). TNM stage 3, T4, N2-N3, and patients who did not receive neoadjuvant chemotherapy had higher recurrences (p = 0.016, p = 0.004, p = 0.036, p = 0.005). Furthermore, the number of cycles of adjuvant treatment in patients with recurrence was statistically significantly higher than in patients without recurrence (p = 0.007).

In the multivariate Cox regression Analysis of relapse risk factors, the most statistically significant findings revealed a 3.3-fold higher risk for individuals with positive surgical margins and a 4.7-fold higher risk for those with vascular invasion compared to their counterparts without these conditions (p = 0.003, p < 0.001) (Table 2).

**Table 2:** Univariate and multivariate cox regression Analysis of relapse risk factors.

	**Univariate Analysis**			**Multivariate Analysis**		
	P	HR	95 % CI Lower-Upper	P	HR	95 % CI Lower-Upper
Age	0,341	1021	0,978–1067			
Positivesurgicalmargin(Ref:Negative)	<0,001	4430	2178–9010	0,003	3292	1488–7279
Grade (Ref:Well-differentiated)	0,672					
Moderately differentiated	0,610	0,732	0,221–2426			
Poorly differentiated	0,930	0,946	0,275–3262			
Diagnosis CEA	0,014	1014	1003–1025			
Diagnosis CA19.9	0,134	1001	1000–1002			
Diagnosis CA125	0,030	1030	1003–1057			
Neural invasion	0,001	3170	1618–6210			
Vascular invasion	<0,001	5173	2490–10,748	<0,001	4735	2248–9973
Metastaticlymph node	<0,001	1045	1022–1068			
PLNR	<0,001	4459	1961–10,136			
TNM (Ref:Stage 0–1)	<0,001					
Stage 2	0,559	1550	0,357–6741			
Stage 3	0,032	4765	1146–19,801			
Stage 4	0,002	12,651	2529–63,288			
N (Ref:0)	0,001					
N(1)	0,117	1914	0,850–4310			
N(2)	0,013	2912	1255–6757			
N(3)	<0,001	4390	2090–9223			
HER-2 positive (Ref:Negative)	0,071	1951	0,944–4031			
ECOG (Ref:0)	0,005					
ECOG(1)	0,035	1890	1046–3414			
ECOG(2)	<0,001	3117	1660–5854			
ECOG(3–4)	0,976	0,000	0,000-.			
NeoadjuvantCT	0,273	0,670	0,327–1372			

**CT**: Chemotherapy**ECOG**: Eastern cooperative oncology group.

Surgical margin positivity, poorly differentiated carcinoma, elevated PLNR, and ECOG 2 performance status were identified as statistically significant risk factors associated with worse survival in the multivariate Cox regression Analysis (p = 0.002, p = 0.005, p = 0.005, p = 0.030, p < 0.001) (Table 3).

**Table 3:** Univariate and multivariate cox regression Analysis of mortality risk factors.

	**Univariate Analysis**			**Multivariate Analysis**		
	P	HR	95 % CI Lower-Upper	P	HR	95 % CI Lower-Upper
Age	0.050	1.035	1.000–1.071			
Positivesurgicalmargin(Ref:Negative)	<0.001	4.707	2.540–8.722	0.002	5.003	1.775–14.102
Location (Ref:Esophagogastricjunction)	0.091					
Grade (Ref: Well-differentiated)	0.004			0.005	2.438	1.316–4.519
Moderately differentiated	0.426	0.613	0.183–2.047			
Poorly differentiated	0.470	1.553	0.470–5.133			
Diagnosis CEA	0.048	1.011	1.000–1.022			
Diagnosis CA19.9	0.001	1.002	1.001–1.003			
Diagnosis CA125	0.071	1.016	0.999–1.035			
Neural invasion	<0.001	2.993	1.708–5.243			
Vascular invasion	<0.001	4.844	2.627–8.932	0.058	2.097	0.974–4.514
Metastaticlymph node	<0.001	1.061	1.042–1.080			
PLNR	<0.001	10.796	5.438–21.43	0.030	3.513	1.127–10.952
TNM (Ref:Stage 0–1)	0.000					
Stage 2	0.996	1.003	0.295–3.406			
Stage 3	0.008	4.789	1.494–15.354			
Stage 4	0.002	8.755	2.256–33.985			
N (Ref: N0)	<0.001					
N(1)	0.264	1.507	0.734–3.092			
N(2)	0.012	2.586	1.227–5.447			
N(3)	<0.001	6.362	3.401–11.902			
HER-2positive (Ref:Negative)	0.445	1.335	0.637–2.797			
ECOG (Ref: ECOG 0)	<0.001			0.004		
ECOG(1)	<0.001	3.232	1.899–5.500	0.068	2.006	0.949–4.242
ECOG(2)	<0.001	5.679	3.212–10.041	<0.001	4.063	1.899–8.692
ECOG(3–4)	0.002	5.252	1.808–15.253	0.981	0.000	0.000
NeoadjuvantCT	0.037	0.435	0.199–0.950			

**CT**: Chemotherapy **ECOG**: Eastern cooperative oncology group**PLNR**: Positive lymph node ratio.

**Fig. 1:**
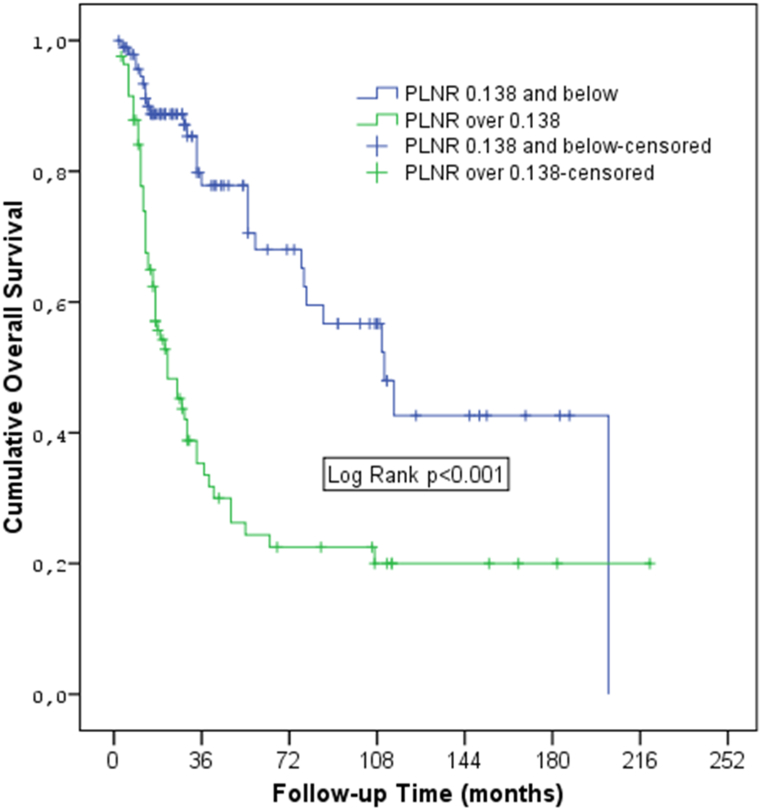
Kaplan–Meier estimates of overall survival.

The cut-off score for PLNR was found to be 0.138 by ROC analysis. Kaplan-Meier curves supported by the log-rank test revealed that the median OS was 22 months in those with a PLNR score above 0.138 and 111 months in those with a PLNR score of 0.138 and below (p < 0.0001, figure). The cut-off value for recurrence-free survival was 0.042. PLNR above 0.042 was significant regarding recurrence (p < 0.0001).(see Fig 1)

## 5 Discussion

Previous reports emphasize the pivotal role of lymph node status as the foremost prognostic factor for recurrence and survival in operated GC patients [13]. The number of lymph nodes dissected is influenced by factors such as surgeon preference, type ofoperation, pathologic evaluation, patient characteristics, and tumor subtype. Insufficient or excessive lymph node dissection may lead to stage migration, potentially resulting in misinterpretations, such as understating the actual stage of the disease. TNM staging remains the predominant system for assessing prognosis, but the optimal number of lymph nodes examined remains controversial. The latest AJCC guidelines recommend dissecting a minimum of 16 lymph nodes to ensure accurate nodal assessment in gastric cancer. Nevertheless, opting for extended lymph node dissection in elderly patients may lead to heightened intraoperative blood loss, an elevated risk of complications due to prolonged operation time, and delayed wound healing post-surgery. Extended lymph node dissection may increase morbidity without providing a noticeable overall survival benefit [8,14]. D2 lymph node dissection remains the standard for patients with good performance status [15]. Standard D2 lymph node dissection may not be possible in elderly patients withcomorbidities. When a small number of lymph nodes are dissected, it will be difficult to predict the patient's prognosis. In this case, PLNR contributes to predicting OS [16]. PLNR has emerged as an independent prognostic factor for GC that is not affected by the total number of lymph nodes.

Numerous alternative PLNR systems, distinct from the traditional TNM stage, have been proposed in the literature, often categorized into 2, 3, 4, or more groups [9]. However, the lack of standardization and the practical challenges in applying these diverse groupings led us to employ a single cut-off value in our study. Different PLNR cut-off values have been suggested in various studies [17,18]. For instance, a study by Durval R. Wohnrath et al. identified a PLNR cut-off value of 0.11 through ROC analysis for survival in 398 operated GC patients. Patients with a PLNR below 0.11 exhibited a median survival of 58 months, whereas those with a PLNR above 0.11 had a median OS of 14 months [10]. Another study by Kano K et al. found a PLNR cut-off value of 0.12 in a cohort of 95 patients who underwent gastrectomy [19]. In our study, one-fourth of the patients underwent D2 dissection. Our analysis revealed no significant difference in recurrence and OS between D1 and D2 dissection.

In our investigation, multivariate analysis identified surgical nerve positivity, poorly differentiated carcinoma, PLNR, and an ECOG 2 performance status as adverse prognostic factors for survival. Our study revealed a critical cut-off value of 0.138 for PLNR in relation to OS, with higher PLNR values associated with worse survival outcomes. In particular, a median OS of only 22 months was observed for PLNR above 0.138, which contrasted with a significantly longer median OS of 111 months for PLNR below 0.138. The PLNR cut-off value for recurrence-free survival was calculated as 0.042. A PLNR higher than 0.042 indicates a high probability of recurrence. We believe that PLNR will be very practical and efficient in predicting prognosis in GC patients.

Our study's limitations include that, given the frailty and potentially shorter life expectancy in the elderly patient cohort, surgical intervention may not have been performed in some cases. Consequently, the survival results observed in our study may have been higher than expected.

Our objective is not to assert the superiority of PLNR over the traditional TNM staging system but rather to investigate whether it represents a novel and practical parameter for predicting survival in conjunction with already established prognostic indicators. Notably, as the PLNR system does not encompass the T stage, it cannot directly substitute for the comprehensive information TNM staging provides.

Subsequent investigations involving a larger patient cohort undergoing limited lymph node dissection can establish standardization for PLNR in clinical practice, offering hope for improved survival outcomes in GC patients.

## 6 Conclusion

Our findings suggest that PLNR emerges as an independent predictor of survival and recurrence in patients undergoing resection for GC. This information may guide treatment decisions for elderly patients with GC and potentially improve their outcomes. However, it is essential to note that the PLNR system does not include the T stage. Therefore, it cannot be a direct substitute for the comprehensive information TNM staging provides. It will help predict prognosis in GC patients undergoing surgery when adequate lymph node dissection is impossible.

## Ethicalapproval

The Health Sciences Ethics Committee approved this study of ManisaCelal Bayar University Faculty of Medicine with the decision dated March 15, 2023 and No. 1745.

## Funding

None.

## Patients' informed consent

Due to the study's retrospective nature, patients' explicit consent was not required.

## Availability of data and materials:

The data sets used and analyzed during the current study are available from the corresponding author upon reasonable request.

## Data availability statement

Data is openly available in a public repository.

## CRediT authorship contribution statement

**Omer Acar:** Writing – review & editing, Writing – original draft, Visualization, Validation, Supervision, Software, Resources, Project administration, Methodology, Data curation, Conceptualization. **Onur Yazdan Balcik:** Formal analysis. **Muslih Urun:** Investigation, Data curation. **Tugay Avci:** Resources, Project administration, Data curation. **Mustafa Sahbazlar:** Software, Resources, Project administration, Methodology, Investigation. **Atike Pinar Erdogan:** Investigation, Formal analysis, Conceptualization.

## Declaration of competing interest

The authors declare that they have no known competing financial interests or personal relationships that could have appeared to influence the work reported in this paper.
